# *In Vivo* MicroCT Monitoring of Osteomyelitis in a Rat Model

**DOI:** 10.1155/2015/587857

**Published:** 2015-04-29

**Authors:** Vincent A. Stadelmann, Inga Potapova, Karin Camenisch, Dirk Nehrbass, R. Geoff Richards, T. Fintan Moriarty

**Affiliations:** AO Research Institute Davos, Clavadelerstrasse 8, 7270 Davos-Platz, Switzerland

## Abstract

Infection associated with orthopedic implants often results in bone loss and requires surgical removal of the implant. The aim of this study was to evaluate morphological changes of bone adjacent to a bacteria-colonized implant, with the aim of identifying temporal patterns that are characteristic of infection. In an *in vivo* study with rats, bone changes were assessed using *in vivo* microCT at 7 time points during a one-month postoperative period. The rats received either a sterile or *Staphylococcus aureus*-colonized polyetheretherketone screw in the tibia. Bone-implant contact, bone fraction, and bone changes (quiescent, resorbed, and new bone) were calculated from consecutive scans and validated against histomorphometry. The screw pullout strength was estimated from FE models and the results were validated against mechanical testing. In the sterile group, bone-implant contact, bone fraction, and mechanical fixation increased steadily until day 14 and then plateaued. In the infected group, they decreased rapidly. Bone formation was reduced while resorption was increased, with maximum effects observed within 6 days. In summary, the model presented is capable of evaluating the patterns of bone changes due to implant-related infections. The combined use of longitudinal *in vivo* microCT imaging and image-based finite element analysis provides characteristic signs of infection within 6 days.

## 1. Introduction

Implant-related osteomyelitis is one of the most feared complications associated with the surgical placement of orthopedic devices such as prosthetic joints and fracture fixation devices. These infections are often characterized by bacterial biofilm formation on the implanted device, eventually leading to osteolysis of adjacent bone and ultimately possible implant failure [1]. Typically, patients with an implant-related bone infection present with symptoms such as fever, pain, and swelling of the operated limb, all of which occur before radiographic evidence of infection (such as osteolysis) is detectable [2]. Early diagnosis of infection is crucial for optimal management of implant-related bone infections as infections may be successfully treated by antibiotics alone if detected within 3 weeks [3, 4]. However, once osteolysis is observed radiologically, the infection may have already entered a chronic phase and surgical intervention is required to remove the biofilm covered implant and debride all necrotic and infected bone [5].

Modern imaging techniques, such as conventional radiography, magnetic resonance imaging (MRI), and computed tomography (CT), provide three-dimensional (3D) images of the bone structure. These imaging techniques are very powerful, but of more value in the later stages of the infection once significant structural changes are apparent. The gold standard for diagnosing infection has therefore remained the invasive sampling of tissue biopsies for conventional bacterial culture and histological analysis [2–4], despite recent advancements in functional imaging techniques such as FDG-PET/CT [6].

One reason CT imaging has not been widely adopted for the diagnosis of infection may be that there is no known specific feature that distinguishes early stage bone infection at a time early enough to impact upon treatment. Even high-resolution CT scans are only capable of detecting bone osteolysis after an infection has resulted in significant bone damage [4]. Microcomputed tomography (microCT), however, has been shown to reveal bone structures with high precision in laboratory animals [7, 8] and has been widely adopted to monitor the progression of bone structural changes at multiple time points in the same living animal [9, 10]. A benefit of this technique is that it imposes minimal burden upon the animal and with sequential images in the same subject can reduce the numbers of animals required for experimental study [3, 4, 11, 12]. Furthermore, by combining microCT images from subsequent time points, it is possible to compute histomorphometric indices, such as bone formation and resorption rates, noninvasively [5, 13, 14]. We have recently demonstrated that* in vivo* microCT can also be used to quantify peri-implant bone adaptation and implant fixation noninvasively, with minimal influence on image quality by metal artifacts [6, 15]. In this study, we explore* in vivo *microCT to characterize the progression of implant-related osteomyelitis in a novel implanted rat tibia model. A correlation is shown between the microCT data with quantitative* ex vivo* bacteriology,* postmortem* histology, and biomechanical testing. Thus, this serves as a model for studies monitoring the progression of implant-related osteomyelitis in a living animal without requiring animal euthanasia at every time point of interest. Characterizing these changes might help develop diagnostic approaches that would focus on patterns of early stage infection rather than searching for signs of established infection. Looking forward, this technique may be applicable to a wide range of research studies including antimicrobial interventions and implant performance studies.

## 2. Materials and Methods

### 2.1. Study Design

**Figure 1:**
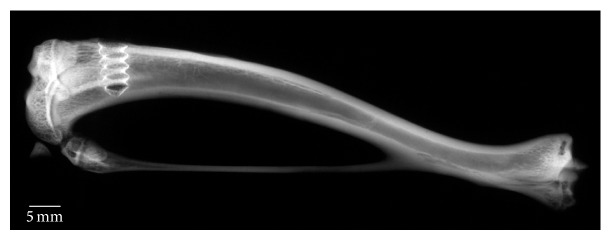
The titanium-coated PEEK screw was placed unicortically in the cancellous part of the right proximomedial tibia (contact radiography, entire specimen [full thickness]).

This study involved a rat model wherein one control (sterile) or bacteria-colonized (*Staphylococcus aureus*) implant was placed in the cancellous bone of the right tibia (Figure 1). The animals were scanned with* in vivo* microCT at predetermined intervals over 28 days. The scan data was used to evaluate bone changes around the screw and to simulate pullout tests with finite element (FE) analysis. After euthanasia, half of the animals were submitted for biomechanical measurement of screw pullout forces to calibrate the FE simulations. Subsequently, these screws underwent quantitative bacterial culture to measure the number of bacteria attached. The remaining half of the samples were fixed, embedded, and sectioned for histological confirmation of infection and to correlate with microCT scans.

### 2.2. Animals

Twenty-four female Wistar rats (weight: 276 ± 18 g; age: 15.5 ± 2 weeks; Charles River, Germany) were used in this study. All procedures were approved by the Graubünden Animal Commission and performed in accordance with the Swiss Animal Protection Law in an AAALAC approved facility. The rats were randomly assigned to one of the two experimental groups: (1) control group receiving sterile implant (*n* = 12) or (2) infection group receiving a bacteria-colonized implant (*n* = 12). For the whole duration of the study, the rats were kept in group-housing (3-4 rats per cage) at 12 hours light/dark cycle in a climate controlled room and given* ad libitum* access to water and food (Alleinfuttermittel für Mäuse und Ratten, Provimi Kliba, Kaiseregg, Switzerland). No antibiotic agents were administered to the rats at any time.

### 2.3. Implants

Custom-made screws (length 5.8 mm, diameter 2.1 mm) were machined from medical grade polyetheretherketone (PEEK, Invibio Biomaterial Solutions, Lancashire, United Kingdom). The threaded portion of the screw was coated by 30 *μ*m thick rough titanium (Ti) coating applied by vacuum plasma spraying (Medicoat AG, Mägenwil, Switzerland). Before use, the Ti coated screws were cleaned by ultrasonication (15 minutes each) in a series of propanol (Sigma-Aldrich, Buchs, Switzerland), 70% ethanol (Sigma-Aldrich), and ultrapure water 113 (MilliQ 18.2 Mcm). All screws were steam sterilized for 20 minutes in an autoclave at 134°C before use.

### 2.4. Bacteria

*Staphylococcus aureus* JAR 06.01.31 was used as the test microorganism. This is a clinical isolate, cultured from a patient with prosthetic joint infection [7, 8, 16]. The strain was maintained in 20% glycerol at −80°C and, when required, grown on a blood agar plate to ensure purity. A single colony was then subcultured into 20 mL prewarmed tryptic soya broth (TSB, Oxoid, Basel, Switzerland) and incubated overnight in 37°C ambient air and shaken at 100 rpm. After overnight growth, the bacterial culture was twice centrifuged at 2500 g for 10 minutes, and the bacteria pellet resuspended in 20 mL PBS each time. The bacterial suspension was then sonicated for 3 minutes and the optical density adjusted to 0.50 at 600 nm, using a spectrophotometer (Multiskango, Thermo Fisher Scientific, Reinach, Switzerland). Three hundred microliters of the bacteria suspension (approximately 3.3 × 10^7^ colony forming units per mL, CFU/mL) were dispensed into a 96-well flat bottom dish. The threaded portion of the screw was submerged within the suspension and incubated at room temperature for 20 minutes to allow bacteria to adhere to the surface. The screws were then removed from the bacterial suspension and allowed to air-dry in a laminar flow hood for 5 minutes. The bacteria-colonized screws were aseptically transported to the surgery unit and inserted into rats within 2 hours.

Quantitative culture of adherent bacteria on identically prepared screws was performed to estimate the number of bacteria given to the animals. These screws were submerged in 10 mL of sterile PBS, vortexed for 20 seconds, and subsequently placed in a sonicating water bath operating at a frequency of 40 kHz for 3 minutes to remove adherent bacteria before a final vortex for 20 s. The sonicates were immediately serially diluted in sterile PBS and 200 *μ*L aliquots spread onto blood agar (Oxoid, Basel, Switzerland) plates for quantitative bacterial culture.

### 2.5. Surgery

All surgeries were performed under inhalation by placing the rats in an induction box with 3% isoflurane (Baxter AG, Volketswil, Switzerland) in 100% oxygen and maintained with around 2% isoflurane in 100% oxygen using a rodent face mask at 500 mL/min oxygen inhalation. Buprenorphine (Temgesic, Rechitt Benckiser, Wallisellen, Switzerland) was given subcutaneously (0.01 mg/kg body weight) for premedication and 2 drops of Articaine (Rudocain, Streuli Pharma AG, Uznach, Switzerland) were given locally during the surgery. Postoperative pain management consisted of Buprenorphine (0.01 mg/kg body weight) s.c. 12 and 24 hours post-op and Paracetamol (Dafalgan Syrup, Bristol-Myers Squibb, Baar, Switzerland) in drinking water (2 mg/mL) for 5 and 28 days postoperatively for control and infected animals, respectively.

For the surgical intervention, the rat was placed in dorsal recumbency and the skin over the right tibia was aseptically prepared. A 1 cm skin incision was then made on the proximolateral aspect of the right tibia. The tibial growth plate and insertion of the patella ligament were then identified on the medial side of the tibia. A unicortical hole was drilled 2 mm distal to the growth plate in the medial tibia using a 1.8 mm diameter drill bit (#310.508, Depuy Synthes, Zuchwil, Switzerland). The drill hole was then tapped with a custom-made stainless steel tap (2 mm outer/1.2 mm inner core diameter). A sterile or a colonized screw was then inserted in the hole for the control and the infected group, respectively. The fascia and the skin were closed in two layers using absorbable suture material (Monocryl and Vicryl rapid, Ethicon Inc., Cincinnati, USA; sizes 6-0 and 5-0, resp.). To prevent hypothermia, the rat was placed on a heated mat for the whole duration of the surgery and under infrared light for the recovery period.

### 2.6. *In Vivo* Microcomputed Tomography (MicroCT)

The evolution of bone structure and bone-implant contact was assessed using* in vivo* microCT (VivaCT40, Scanco Medical AG, Brüttisellen, Switzerland). The animals were scanned directly after surgery and at 3, 6, 9, 14, 20, and 28 days postoperatively. The animals were placed in the standard rat holder, provided by the manufacturer, under 2% vol. isoflurane anesthesia. The right tibia was extended to fit in the field of view and the ankle was fixed securely with masking tape. A 10 mm region, centered on the implanted screw, was scanned with a ø25.6 mm field of view, at 70 kV tension, 114 *μ*A current, 204 ms integration time, and 1000 projections per scan. This protocol has been shown to have no impact on rat bone [9, 10, 12, 15]. The projections were then reconstructed across an image matrix of 2048 × 2048 pixels with an isotropic voxel size of 12.5 *μ*m. Scans showing evidence of severe artifacts were excluded.

### 2.7. Image Processing

After reconstruction, noise in the scan data was Gaussian filtered (sigma 0.8, support 1). For each animal, the scan series were aligned to the postoperative scan using rigid registration, with a minimum correlation coefficient of 0.8, convergence criterion of 1 × 10^−5^, and maximum 2000 iterations [17, 18]. Scans that would not satisfy these registration criteria were excluded. The registered scans were then segmented with bone threshold set at 515 mgHA/cm^3^ and titanium coating at 1500 mgHA/cm^3^. The segmented coating was dilated by one voxel to compensate for partial volume effects.

Two regions of interest (ROIs) were used for image analysis: the implant surface (ROI1) and peri-implant bone (ROI2). Both regions were first defined around a virtual screw by successive dilations of 75 *μ*m and 1 mm from the screw surface, for ROI1 and ROI2, respectively. Both ROIs were limited to the threaded part of the screw to limit variations due to differences in insertion depths. This virtual template was used to generate the actual ROIs in the scan series in a reproducible fashion. For each scan, the virtual screw coating was registered to the segmented coating and the resulting transformation was applied to the ROI template to generate the final ROIs for analysis.

Bone fraction was computed from the segmented images to evaluate bone-implant contact (BIC) in ROI1 and bone fraction (BV/TV) in ROI2. Afterwards, quiescent, resorbed, and newly formed bones were evaluated from differences between registered subsequent images within ROI2, as described by Waarsing et al. [13].

All image processing and analysis were performed with image processing language (IPL, Scanco Medical AG).

### 2.8. Finite Element Modeling

**Figure 2:**
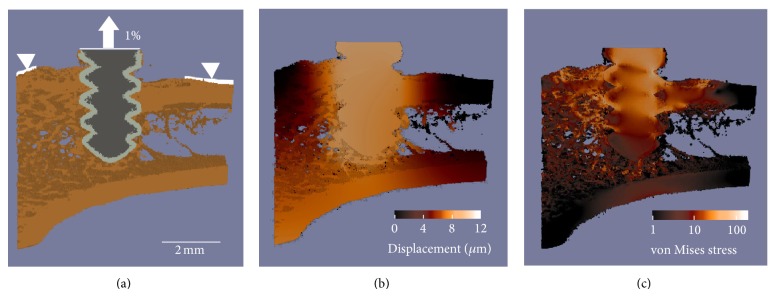
(a) Microfinite element pullout model. The screw head is subjected to a 1% displacement and bone surface 2 mm away from the screw is fully constrained. Examples of computed (b) von Mises stress distribution and (c) computed displacement field.

The scan data was transformed into microfinite element (microFE) models to evaluate the screw pullout strength at all time points, as described previously [5, 15]. In brief, the segmented images were prepared for linear elastic analysis using the voxel conversion approach [19]. The isotropic Young's modulus was set at 3.6 GPa for PEEK, 70 GPa for Ti, and 6.8 GPa for bone; Poisson's ratio was set at 0.3 for all materials. Boundary conditions were applied to simulate uniaxial pullout of the screw, with 1% axial displacement on the screw head. The upper surface of the bone was fully constrained in all directions, except for bone elements within 2 mm of the screw axis, which were unconstrained (Figure 2(a)). The models were then solved with Faim 6.0 (Numerics88 Solutions Ltd, Calgary, Canada) on a desktop workstation (MacPro, Apple Inc., Cupertino, CA, USA) for pullout stiffness (N/mm) and failure load (N) estimated with the “Pistoia criterion” [6, 20, 21] (Figures 2(b) and 2(c)).

### 2.9. Euthanasia and Tissue Retrieval

Upon completion of the 28-day observation period, the animals were euthanized with an overdose of CO_2_. The tibiae containing the screws were immediately harvested. Six samples from both the infected and sterile groups were used for histological evaluation and six of each group were used for mechanical testing followed by quantitative bacteriology. Specimens for mechanical testing were frozen at −20°C and thawed before testing in a single batch.

### 2.10. Histological Processing and Histomorphometric Analysis

Contact radiographs were taken of the entire fresh specimens using a cabinet X-ray system (Faxitron 43855A, Faxitron Bioptics, Tucson, AZ, USA). The specimens were then fixed with 70% methanol for two weeks, with a fresh solution change after one week, after which the specimens were dehydrated in an ascending series of ethanol (70, 96, and 100%), changing the solutions twice weekly. They were transferred to xylene for five days and then embedded in methyl methacrylate (Sigma-Aldrich). Serial sections of 200 *μ*m thickness were cut through the longitudinal axis of the implants using an annular blade saw (Leica SP1600, Leica Biosystems, Heerbrugg, Switzerland). Two consecutive sections were glued onto opaque plexiglass slides with cyanoacrylate, ground, and polished down to 100 *μ*m using a microgrinding device (400CS, EXAKT, Norderstedt, Germany). One section from each specimen was surface stained with Giemsa-Eosin for tissue morphology and the other with Brown-Brenn for bacterial staining.

Qualitative histopathological analysis of the stained slides was performed on all samples using a light microscope (BX40, Olympus AG, Volketswil, Switzerland). Microscopic images of the stained slices were obtained with a light microscope (Axioplan Imager 2, Zeiss GmbH, Jena, Germany) using a 1.25x lens and a high-resolution camera (Axiocam HRc, Zeiss). Bone-implant contact was manually defined on overview images of Giemsa-Eosin stained sections using histomorphometric software (Axiovision 4.8, Zeiss). Bone-implant contact was determined by interactive measurements on each digital image as the ratio of bone-implant contact length to implant circumference using a custom macro (KS400, Zeiss).

### 2.11. Mechanical Testing

The specimens were thawed prior to mechanical testing and the remaining soft tissues were removed manually using sterile surgical dissection instruments. A custom-made clamping system [7–10, 22] was used to link the screw head to the testing machine plate, and a rigid steel plate with a 4 mm diameter hole was used to retain the specimen in the fixture system. The fixture system was attached to a 1 kN load cell on the testing machine (Instron 4302, Instron, MA, USA). The screw was pulled out until failure using a displacement rate of 1 mm/min. The pullout force and displacement were sampled at 10 Hz. Ultimate load (N), yield load (N), and stiffness (N/mm) were calculated from the load-displacement curves with Matlab (The MathWorks Inc., Natick, MA, USA). Experimental stiffness and yield force were correlated with microFE stiffness and failure load at day 28 using simple linear regression analyses. The models parameters were used to calibrate the microFE stiffness and failure load at all previous time points.

### 2.12. Bacteriology

After pullout, the screws and bones were collected in separate, sterile containers with 5 mL PBS. The number of bacteria associated with the screw was determined by sonicating the screws in 5 mL PBS for 3 minutes and vortex mixing for 10 seconds before performing serial dilution and viable counts on 5% horse blood agar (Oxoid, Basel, Switzerland). The entire tibia from each animal was then mechanically homogenized (Omni Tissue Homogenizer and Hard Tissue Homogenizing tips, Omni International, Georgia, USA) to give an even suspension. The quantity of bacteria associated with bone was quantified by serial dilution and viable counts on blood agar. All agar plates were incubated for 24 hours at 37°C and all growth checked for contamination or signs of coinfection. Identification of the bacteria growing in culture positive samples was performed for at least one colony from each culture positive animal, by a* S. aureus* latex agglutination assay (Staphaurex, by Remel, Thermo Scientific, Zurich, Switzerland).

### 2.13. Data Analysis

Data is reported as mean ± SD unless stated otherwise. Analysis of variance (ANOVA) with repeated measures was used to determine statistical significances. When appropriate, Bonferroni post hoc tests were performed to compare group means. Correlations between experimental and numerical data were done with linear regression analysis, and Pearson's correlation coefficient is reported. All statistics were performed with R [9–12, 23].

## 3. Results

### 3.1. Animal Welfare

The animals recovered quickly after surgery and showed no signs of discomfort. They demonstrated normal behavior and mobility within hours of the interventions. During the 28 days of the experiment, the control animals gained 25 ± 9 g of body weight, consistent with young growing rats, whilst weight change in infected group was more variable, with a mean change of 1 ± 21 g. The difference between the groups was significant (*P* < 0.05).

### 3.2. Bacteriology

The number of bacteria associated with the screw and surrounding bone was quantitatively assessed by performing a total viable count of sonicated implants and homogenized tissue samples. The results revealed that the quantity of bacteria present on the implant upon placement was 5.9 ± 2.4 × 10^5^ CFU/screw. Upon completion of the 28-day observation period, the average number of bacteria cultured from the screw was 1.8 ± 1.6 × 10^6^ CFU/screw. In the surrounding bone, the bacterial numbers reached 5.5 ± 5.7 × 10^5^ CFU. Bacteria were also detected in the surrounding soft tissue, though at lower numbers (2.9 ± 3.7 × 10^5^ CFU). All bacterial growth was confirmed to be* S. aureus *and no other bacterial pathogens were identified based upon colony morphology. Quantitative cultures of the sterile group specimens were all negative.

### 3.3. Histopathology

**Figure 3:**
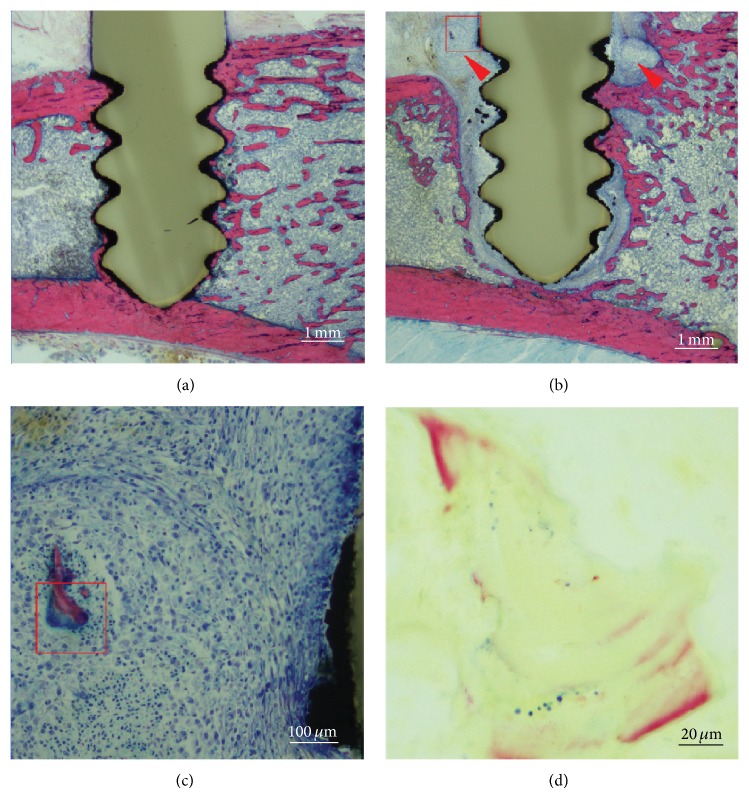
Microphotographs of histological sections. Giemsa-Eosin stained overview of (a) sterile screw showing direct osseointegration of the screw without any signs of osteolysis and (b) an inoculated screw showing loosening, pronounced osteolysis, and massive purulent inflammation with formation of microabscesses (red arrows) partly with a small infected dead bone chip (red box). (c) Magnification of red box showing microabscess around a dead bone chip (stained purple, red box). (d) Magnification of the infected bone chip and Brown-Brenn positive coccoid bacteria.

Evaluations of the stained sections in the control animals revealed a high grade of direct bone contact of the titanium-coated screw at both the cortical and the medullary interface, without any signs of osteolysis (Figure 3(a)). In contrast, the animals receiving colonized screws displayed marked osteolysis, multiple small sequestered bone chips, and massive purulent inflammation. Microabscesses composed of neutrophilic granulocytes, cellular debris, coccoid bacterial colonies, and a fibrous capsule formation around the screw were also observed in all the infected animals after 28 days (Figures 3(b)–3(d)).

### 3.4. *In Vivo *MicroCT

Only 2 out of 168 microCT scans were discarded due to motion artifacts. The discarded scans were not repeated, as it would have required the animals to be reanesthetized. No metal artifacts were observed in the scans despite the use of the 30 *μ*m thick titanium coating. Fifteen scans in the infected groups at later time points were not used for bone evolution analysis, as they did not satisfy the registration criteria. In these scans, the screws were found to slowly migrate in each consecutive scan, making image registration and comparison impossible.

Bone evolution was different between control and infected animals. The sterile screws were rapidly encapsulated by dense bone until day 9 to 14, and by day 28 this new bone was remodeled into a cancellous-like structure, similar to what has been described previously around titanium implants [3, 4, 11–15, 24–26] (Figure 6(a)). Quantitatively, in the control group, BIC and BV/TV showed a rapid increase until day 9, followed by a slow decay/stabilization until day 28. The changes were most prominent between day 0 and day 9, with peak of +89% and +92% in BIC and BV/TV, respectively. Bone formation was increased between days 3 and 9, peaking at 0.34 ± 0.06 mm^3^/day, and resorption stayed constant at 0.11 ± 0.03 mm^3^/day.

**Figure 4:**
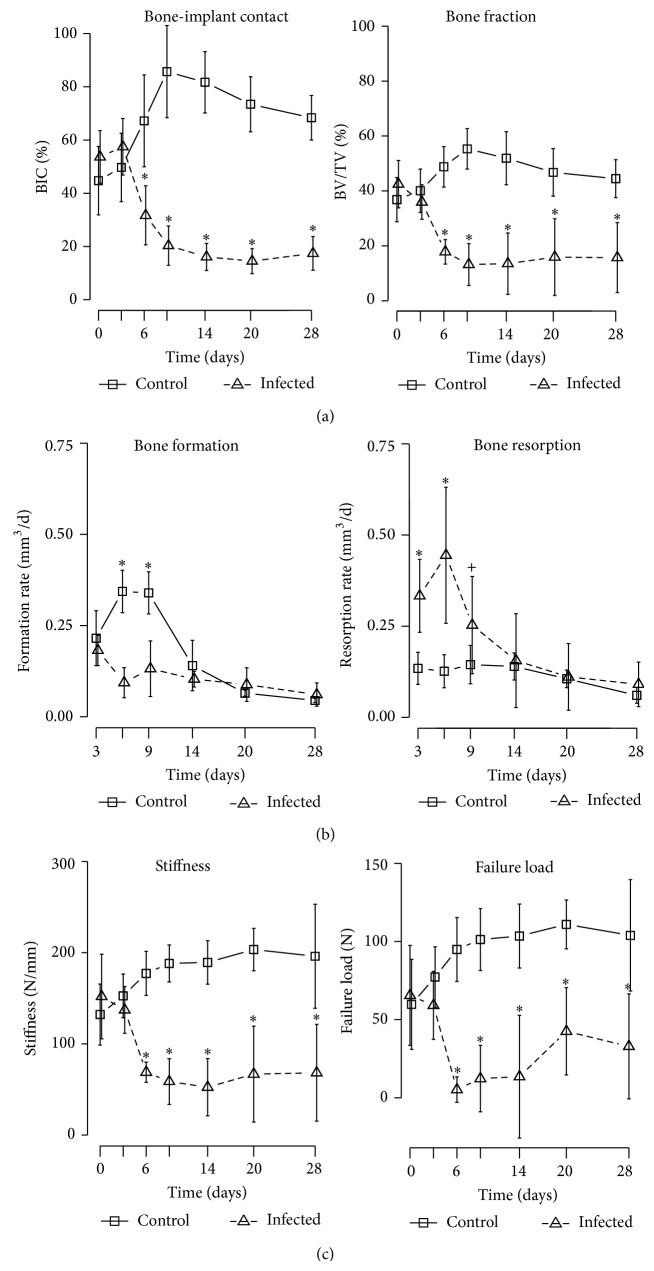
(a) Evolution of bone-implant contact (BIC) in ROI1 and bone fraction (BV/TV) in ROI2 evaluated by* in vivo* microCT. The evolution was significantly different between control and infected animals, with sterile control screws leading to rapid integration and bacteria colonized screws leading to rapid bone loss. (b) Bone formation and bone resorption evaluated by comparing subsequent* in vivo* microCT scans. Infected animals suffer a significantly reduced bone formation between days 3 and 6 post-op and a significantly increased bone resorption until day 9 post-op. (c) Evolution of stiffness and failure load evaluated by microFE simulations. (^∗^ *P* < 0.01, ^+^ *P* < 0.05).

**Figure 5:**
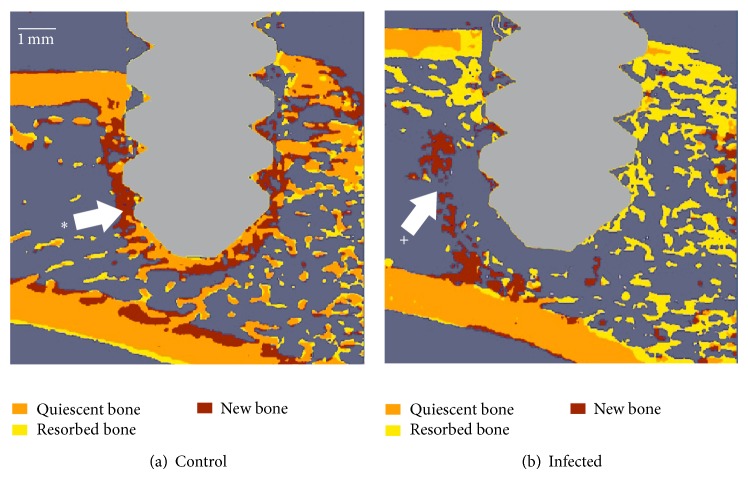
(a) Bone changes around a sterile screw and (b) around an inoculated screw. The images show the computed differences between the scan 9 days post-op and the scan immediately post-op. The sterile screws are encapsulated in new bone (∗). Massive bone loss occurs around inoculated screws. Surprisingly, the formation of a bone wall was observed around the infected area (+).

In contrast, bone around colonized screws was rapidly resorbed by day 6 to 9 and later replaced by fibrous tissue as shown by histology (Figure 5(a)). Surprisingly, new bone formation was observed around the fibrous capsule as early as day 9 and maintained until day 28 (Figure 5(b)). In six out of twelve specimens, the screws had no more bone contact after day 20. The infected animals showed rapid and significant loss until between days 3 and 14. Total BIC and BV/TV losses were −73% and −74%, respectively. The difference compared to control animals was significant from day 6 onward (*P* < 0.01). Both bone formation and bone resorption were altered around colonized screws. Infected animals showed reduced bone formation after surgery and a strong increase in resorption between days 3 and 9, peaking at 0.44 mm^3^/day (Figure 4).

### 3.5. MicroFE

**Figure 6:**
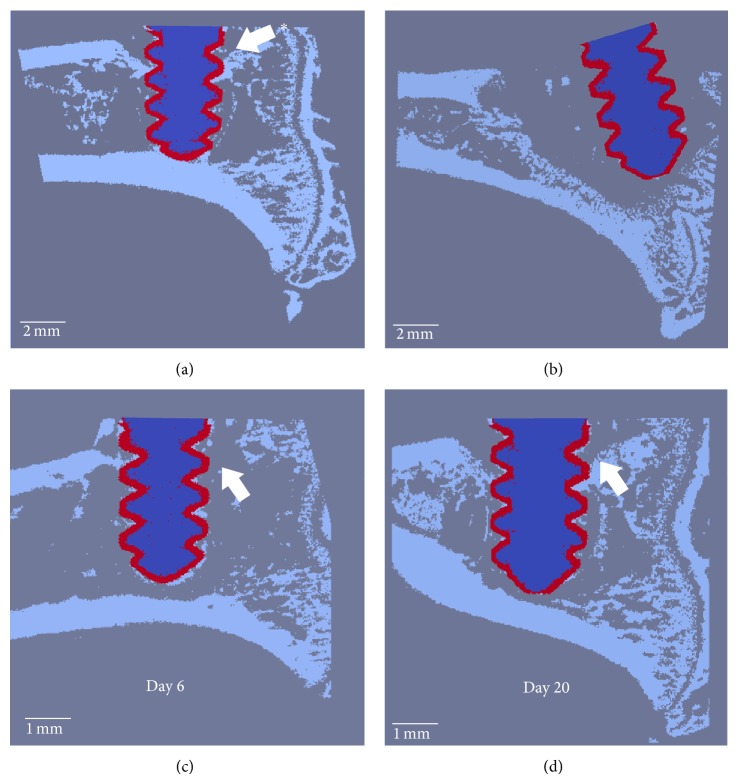
(a, b) Two inoculated specimens showing different conditions at day 28: (a) in this specimen, some bone-implant contact and cortical fixation were preserved at day 28. (b) Total loss of bone-implant contact, peri-implant bone stock leading to a fully loosened screw. (c, d) Example of an animal showing reformation of pseudocortical bone around the screw after a quasicomplete osteolysis: (c) at 6 days, very little cortical bone is left in contact with the screw thread. Pullout force is reduced significantly. (d) At day 20, we observe a neocortical formation and bone-implant contact, restoring partially the screw fixation.

Only the specimens that remained in their original position were used for microFE simulations and mechanical testing. The initial fixation of the screws in both groups was similar, with stiffness of 132 ± 33 and 152 ± 41 N/mm and initial failure load of 60 ± 29 and 68 ± 30 N in the control and infected group, respectively. In the control group, both stiffness and failure load increased between day 0 and 9, by approximately 40% and 85%, respectively, and then plateaued, similar to what has been reported previously for titanium implants [5, 13–15]. In contrast, infected screws showed a rapid decrease in stiffness and failure load between days 0 and 6, by approximately 59% and 98%, respectively (Figure 4). The differences between the two groups were significant from day 6 onward (*P* ≤ 0.01). Some infected animals showed signs of recovery in terms of cortical bone connection: these animals have almost completely lost bone-implant contact at day 6, but a cortex-like structure is reformed by day 20 (Figures 6(c) and 6(d)), which may explain the small increase in mechanical properties between days 14 and 28.

### 3.6. Validation of MicroCT and MicroFE Evaluations against Postmortem Experimental Data

**Figure 7:**
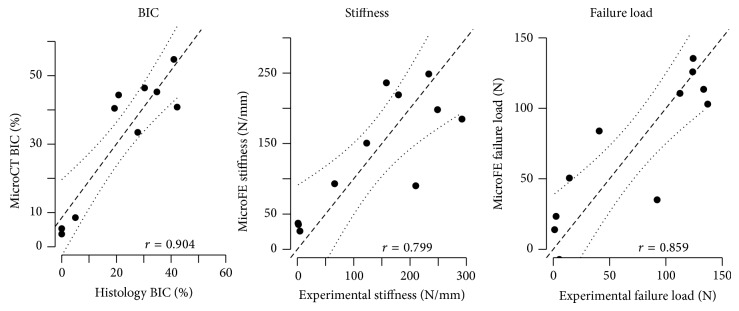
Validation of the microCT-derived outcomes by correlation against experimental data for BIC, stiffness, and failure load. (Dashed line is the linear regression; dotted is the 95% confidence interval).

At day 28, BIC evaluated from* in vivo* microCT correlated significantly with BIC evaluated from histological stained sections (*P* < 0.001), with *r* = 0.89. Numerical simulations of pullout tests correlated significantly with experimental pullouts (*P* < 0.01), with correlation coefficients *r* = 0.80 and 0.86 for stiffness and failure load, respectively (Figure 7). This is an important result for the present study, as it validates the nondestructive virtual analyses of all the other time points.

Finally, to evaluate the reproducibility of our ROI template approach, a postoperative scan was rotated randomly in space seven times, and each position was analyzed independently with this procedure. We found variations of 0.2% relative standard error on BIC and BV/TV and 1.5% for new/resorbed bone volumes. These errors result from the various image transformations before segmentations and from the limited precision of the registration procedure for ROI definitions.

## 4. Discussion

*In vivo *microCT has been used previously to monitor bone evolution adjacent to newly placed sterile implants. With the present study, we apply this technique, for the first time, to a model of implant-related osteomyelitis.* In vivo* microCT imaging of implant-related bacterial infections enables an accurate assessment of bone formation and resorption to be monitored longitudinally and nondestructively in the same animal throughout the progression of the infection. Moreover, in contrast with existing models, which focus on the development of the bacterial population through bioluminescence imaging alone [6, 15, 27] or combined with low-resolution CT [7, 8, 16, 28], our model provides precise data on the bone-implant contact and bone microstructure and allows estimating the implant fixation strength.

The radiolucent screw model developed in this study proved to be reliable for longitudinal monitoring of implant-related infections in live animals. Using a PEEK screw placed proximally in the tibia, the image quality can be maximized with high resolution and low signal-to-noise ratio. This approach resulted in only 1.2% of the scans discarded due to image artifacts, mostly motion artifacts due to animal spasms during the scan. This is a significant progress compared to our previous study with pure titanium implants, for example, where 15% of scans were discarded [15]. The results of the present study with PEEK screws and our previous work with titanium screws display similar behavior: in sterile conditions most of the BIC, BV/TV, and strength gains occur within the first 9 to 14 days post-op [9, 10, 12, 15]. Finally, the correlations between microCT-derived data and experimental data (histomorphometric and mechanical) support the value of this approach for research into peri-implant bone loss due to infection. Importantly, this approach minimizes the number of animals required, particularly in comparison with single time point studies requiring animal sacrifice for quantifiable outcome measures.

In this study, we have monitored the progression of osteolysis surrounding a* S. aureus*-colonized implant over time in a rat model. While control specimens showed a normal osseointegration,* in vivo* microCT revealed a rapid bone loss adjacent to the colonized screws, commencing at 3 days postoperatively. Bone evolution analysis showed that both bone formation and resorption were affected by the bacteria: while control animals show a spurt of bone formation between days 6 and 9 post-op, this bone growth was not observed at any time in infected animals. Bone resorption was stable in control animals, but in infected animals it increased in the first few days and reached a maximum around day 6. The decrease observed after day 6 was probably due to the fact that there was almost no bone left in the ROI after day 9. Looking at the bone formation-resorption net balance, control animals showed a peak of +0.7 mm^3^/d at day 6, while infected animals showed a minimum of −1 mm^3^/d at day 6.

**Figure 8:**
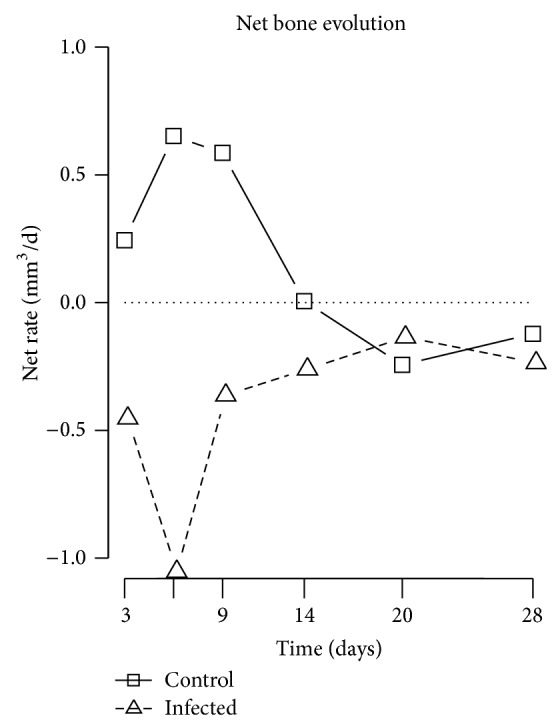
Net bone evolution (formation and resorption rate): control animals show a net bone gain between days 0 and 14, followed by a small loss between days 14 and 28 (remodeling), resulting in an overall gain. In contrary, infected animals show continuous bone loss, with a maximum intensity at day 6.

Previous reports have shed light on the mechanisms of* S. aureus*-induced bone loss.* S. aureus* can induce osteoblastic apoptosis, resulting in decreased bone formation and increased osteoclastic activity through the RANK/RANKL signaling pathway [17, 18, 29]. In comparative terms, ovariectomy, the surgical removal of ovaries, is used in rats to generate massive bone loss for osteoporosis models. Ovariectomy induces a loss of 60% of bone volume in metaphyseal trabecular bone within 4 weeks [13]. This would correspond to an average loss of −0.086 mm^3^/d in our region of interest ROI2. Here, with* S. aureus* infection we see an average loss of −0.42 mm^3^/d, that is, fivefold faster than ovariectomy, with a peak at −1 mm^3^/d at day 6 (Figure 8).

*S. aureus* is a suitable pathogen for the first validation of our technique as it is responsible for approximately 30% of all cases of implant-related bone infections including prosthetic joint and fracture fixation devices [15, 30]. In human patients,* S. aureus *osteomyelitis is associated with a rapid onset of symptoms, caused by a wide array of virulence factors that enable it to survive in the host and induce tissue damage such as osteolysis [19, 31, 32]. Experimental* in vivo* studies using bioluminescent bacteria have revealed that the bacterial load reaches a maximum 3 to 7 days after inoculation [20, 21, 33] leading to acute-phase inflammatory symptoms such as swelling pain and redness, which is very similar to our findings. Bone loss does not become apparent in conventional radiographs until later in the chronic phase, replicating a pattern similar to implant-associated osteomyelitis in human patients. Biooptical imaging of bioluminescent bacteria does not offer potential for clinical diagnosis of infection, as normal bacterial strains are not naturally bioluminescent and thus not detectable by this technique. In contrast, our described CT technique may enable the detection of bacterial infection at a very early stage, by switching focus from the cause of the infection (i.e., bacteria) to the effect (i.e., osteolysis), and does so using equipment that may be feasible for clinical application in the near future.

It is important to note that high doses of bacteria were used in the present study. The seeding inoculum in a modern surgical theater would be significantly less, with estimated bacterial seeding rates for a standard operating theatre during a surgical procedure around 270 bacteria/cm^2^/hr [34]. Reducing the bacterial inoculum to more closely mimic clinical seeding rates would likely result in an infection incidence of 1–5%, and thus 95% of animals may not develop an infection. The advantage of using high loads of bacteria is that all specimens evolve rather similarly, making comparison between groups and statistical analysis possible. If fewer bacteria were inoculated initially, the onset of infections may also be variable over time, inducing much more variability in the data and less statistical power. We consider our inoculum would represent a realistic approach to modeling infection in a manner suitable for experimental studies.

Looking forward, it would be interesting to let the study run longer to observe the long-term host reactions as the infection develops into a more chronic phase. However, the chances of screw migration resulting from extensive reaction around the implant increase significantly with time, and the number of exclusions would become unacceptable. Furthermore,* S. aureus *is an aggressive pathogen, more associated with acute infection where symptoms appear rapidly requiring immediate intervention. Subacute or chronic infections are more typically caused by lower virulence microorganisms such as* S. epidermidis *or* Propionibacterium acnes*. We believe our described model would represent an ideal test bed to monitor implant adjacent bone loss due to these low virulence microorganisms over time, particularly as there is continued controversy regarding the relative contribution of difficulty to isolate microorganisms such as* P. acnes* in loosening of prosthetic devices [35]. Combinations of bacteria could also be tested. Polymicrobial infections represent approximately 20% of all implant-related bone infections [36], which may often include a staphylococcus and other microorganism such as* P. acnes. *Such studies may also require a reduced inoculum and thus enable investigations of bacterial pathogenicity using a bacterial inoculum closer to clinical contamination rates [37]. The present titanium coating, although used clinically as a surface coating for hip prostheses, is not a fully abrasion resistant coating when applied to the small screws used for our study. Evidence of particles eroding from the coating was seen in some CT scans and histological sections. Ti particles are known to induce host reactions leading to aseptic loosening [38]. However, no sign of inflammation was observed around the particles in sterile specimens, and the infections in the infected specimens are so extensive that they would likely override any inflammation purely due to the particles themselves. It is also worth mentioning that image processing of Ti-coated PEEK screws represents a challenge compared to Ti screws. Since PEEK is radiolucent, only the Ti coating is visible and therefore must be used as reference for the registration of the ROI template onto the scans. This requires substantial computation times, up to 6 hours per scan on a dedicated server, to reach the required precision.

In summary, the model presented is capable of evaluating the patterns of bone changes around implant-related infections. The combined use of longitudinal* in vivo* microCT imaging and image-based finite element analysis provides novel data compared to conventional methods. At the same time it reduces the number of animals required to a bare minimum. As an alternative to longitudinal observations of bioluminescent bacteria, the described CT model actually provides superior monitoring of the process of osteolysis adjacent to bacteria-colonized implants. The model could be used to compare treatment options, such as effect of antibiotics, antibacterial coatings, or antiresorbing coatings, on peri-implant bone adaptation.
